# 3D Laser Scanner for Underwater Manipulation

**DOI:** 10.3390/s18041086

**Published:** 2018-04-04

**Authors:** Albert Palomer, Pere Ridao, Dina Youakim, David Ribas, Josep Forest, Yvan Petillot

**Affiliations:** 1 Computer Vision and Robotics Research Institute (VICOROB), Universitat de Girona, 17003 Girona, Spain; pere@eia.udg.edu (P.R.); dina.isaac@udg.edu (D.Y.); josep.forest@udg.edu (J.F.); 2 IQUA Robotics, 17003 Girona, Spain; david.ribas@iquarobotics.com; 3 Ocean Systems Laboratory, Heriot-Watt University, Edinburgh EH14 4AS, UK; y.r.petillot@hw.ac.uk

**Keywords:** 3D, underwater, laser, manipulation, point clouds

## Abstract

Nowadays, research in autonomous underwater manipulation has demonstrated simple applications like picking an object from the sea floor, turning a valve or plugging and unplugging a connector. These are fairly simple tasks compared with those already demonstrated by the mobile robotics community, which include, among others, safe arm motion within areas populated with a priori unknown obstacles or the recognition and location of objects based on their 3D model to grasp them. Kinect-like 3D sensors have contributed significantly to the advance of mobile manipulation providing 3D sensing capabilities in real-time at low cost. Unfortunately, the underwater robotics community is lacking a 3D sensor with similar capabilities to provide rich 3D information of the work space. In this paper, we present a new underwater 3D laser scanner and demonstrate its capabilities for underwater manipulation. In order to use this sensor in conjunction with manipulators, a calibration method to find the relative position between the manipulator and the 3D laser scanner is presented. Then, two different advanced underwater manipulation tasks beyond the state of the art are demonstrated using two different manipulation systems. First, an eight Degrees of Freedom (DoF) fixed-base manipulator system is used to demonstrate arm motion within a work space populated with a priori unknown fixed obstacles. Next, an eight DoF free floating Underwater Vehicle-Manipulator System (UVMS) is used to autonomously grasp an object from the bottom of a water tank.

## 1. Introduction

Autonomous Underwater Vehicles (AUVs) are mostly used for survey missions. Nevertheless, a large number of potential applications require intervention going beyond their current capabilities (e.g., the maintenance of permanent observatories, submerged oil wells, the search and recovery of black-boxes, etc.). Such applications are currently tackled using work-class Remotely Operated underwater Vehicles (ROVs) with a significant cost dominated by the cost of the ship. Advancing towards Intervention Autonomous Underwater Vehicles (IAUVs) may reduce drastically the cost of these operations. Although scientists have significantly advanced the state of the art during the last decade, today’s technology is still far from the capabilities already demonstrated in other robotics fields. For instance, to the best of the authors knowledge, none of the systems reported in the underwater robotics mobile manipulation literature have published experimental results using motion-planning and obstacle avoidance methods, while in other robotic domains (mobile robotics, humanoids, etc.), such techniques are routinely used. Mobile robots have demonstrated the capability to identify objects using depth-RGB images, allowing them to better understand the scene where the manipulation takes place [1,2]. Knowing the surrounding objects, we introduce semantics in the intervention process. It allows understanding, for instance, which objects are graspable and how they should be grasped, bringing the manipulation operation to the next level. Underwater robots are very far from exhibiting those capabilities. The authors think that one of the reasons explaining why underwater mobile manipulators are so immature with respect to land-based robots is due to the lack of proper sensing devices. With the appearance of the Kinect, 3D sensing has drastically reduced in cost, becoming a very popular sensor for mobile manipulation. Unfortunately, it does not work properly underwater, and a clear alternative does not exist yet.

This paper presents a new real-time 3D laser scanner designed to improve the capabilities of the Underwater Vehicle-Manipulator Systems (UVMSs). The scanner is able to provide 3D scans at different resolutions and frequencies (number of scans per second). A method to calibrate the laser-arm system is also proposed, and the complete system is demonstrated in two applications: (1) an eight Degrees of Freedom (DoF) underwater manipulator moving in a water tank populated with a priori unknown obstacles and (2) an eight DoF Intervention Autonomous Underwater Vehicles (IAUVs) grasping an object from the seabed.

The paper is organized as follows. First, a review of the literature is presented in Section 2. Then, Section 3 explains the mechatronics and Section 4 the calibration methods. The experimental results are presented in Section 5 before concluding in Section 6.

## 2. Related Work

The research on autonomous underwater intervention goes back to the early 1990s with the pioneering works of OTTER [3], ODIN [4], UNION [5] and AMADEUS [6], although field demonstration did not arrive until the first decade of the 21st Century. The first fully-autonomous intervention at sea, was demonstrated by ALIVE [7], where a hovering-capable AUV was able to home and dock to a sub-sea intervention panel to open/close a valve. In this project, the panel was a priori known, and the features of the panel were used to compute the relative position of the AUV with respect to it. The first object manipulation from a floating vehicle was achieved in 2009 within the SAUVIM project [8]. It demonstrated the capability of searching for an object whose position was roughly known a priori. The object was endowed with artificial landmarks, and the robot autonomously located and hooked it to a recovery device while hovering. The first multi-purpose object search and recovery strategy was demonstrated in the TRIDENTproject in 2012. First, the object was searched using a down-looking camera and photo mosaic techniques. Next, it was demonstrated how to autonomously “hook” the object to a water tank [9]. The experiment was repeated in a harbour environment using a four DoF arm [10], and later on with a seven DoF manipulator equipped with a three-finger hand [11]. In this project, the object was known, and its position was computed using correspondence between the image and a priori model.

Given the importance of Inspection, Maintenance and Repair (IMR) tasks for the offshore industry, representative tasks usually performed by ROVs, like valve turning and connector plugging or unplugging, have been automated with different approaches. Fixed-base, fully-autonomous valve turning has been demonstrated twice. In [7], a mechanical scanning imaging sonar was used to locate and home to a sub-sea panel using visual servoing techniques for docking the vehicle with two hydraulic grasps. Once docked, a hydraulic seven DoF manipulator was used to open/close a valve that was highlighted with a circle. Similarly, [12] used an active localization strategy based on a sum of Gaussian filter to discover the sub-sea panel with which the AUV should interact [13]. Next, visual servoing methods based on the a priori known appearance of the sub-sea panel were used to autonomously dock the robot into a funnel-based docking station. In this case, no latching mechanism was used. Therefore, to keep the AUV docked to the intervention panel, it was necessary to keep pushing it with a small force until the intervention operation was concluded. This resulted in minor motion of the vehicle with respect to the panel and forced the usage of computer vision techniques to detect the valve handle in order to turn it. In the same work, a first autonomous demonstration of a connector unplugging and plugging operation with a fixed-base was also carried out. In this work, the panel and its texture were known, allowing the vehicle position to be computed from a single image, and an artificial landmark was positioned next to the valve to identify it. The work done by the authors in the PANDORA project demonstrated more a challenging scenario by performing autonomous free-floating valve turning operations on a sub-sea panel using a learning by demonstration paradigm [14]. More recently, a task priority redundancy control approach has been used by the authors for the kinematics control of an UVMS, again to demonstrate the free-floating autonomous valve turning [15]. In these two works, the valve panel was known a priori, providing good robot localization. Moreover, the algorithm had to deal only with the angle of the valve because its position was already known from the panel. Finally, to the best of the authors’ knowledge, none of the state of the art underwater works deal with scenes cluttered with obstacles to avoid while executing the manipulation.

Despite all this work, the lack of Kinect-like underwater sensors has forced the use of computer vision techniques instead of 3D point cloud algorithms for autonomous manipulation. The only available sensors that produce 3D point clouds that could be used in for this purposes are: (1) a Kinect mounted in an underwater housing; (2) stereo cameras; and (3) laser scanners. Although the distortion introduced by the viewport can be corrected when using a Kinect in an underwater housing, the infra-red light that it uses is attenuated too fast, reaching only 20-cm distances [16]. In contrast, stereo cameras can work at longer ranges, up to a few meters, but they are constrained to the existence of features in the observed scene. Without features, the stereo pair cannot reconstruct the environment. Laser scanners overcome such a limitation by using structured light projection. Therefore, they work in more scenarios than stereo cameras. However, laser scanners suffer from a distortion on the reconstructed cloud proportional to the motion of the sensor while it was gathering the scan. Already existing laser scanners normally use a laser plane and a fixed camera, being able to reconstruct a single profile. Often, those scanners are actuated with one DoF, in order to be able to provide a sector scan (commonly mounted on a tripod, or from a landed ROV). Since it takes a significant time to gather a sector scan (a few seconds), although they may be appropriate from mapping, they are not suitable for manipulation, which requires a fast scanning speed. Instead, the scanner proposed here steers the laser beam using a fast mirror providing a significantly faster scanning speed enabling advanced manipulation tasks similar to those shown by land mobile manipulators and/or humanoids such as moving a manipulator without colliding with the environment, detecting and identifying an object and its position or grasping that object in a cluttered environment.

## 3. Mechatronics

In this section, the proposed laser scanner is introduced first, and then, the robotics systems in which it is integrated are presented.

### 3.1. Laser Scanner

**Figure 1:**
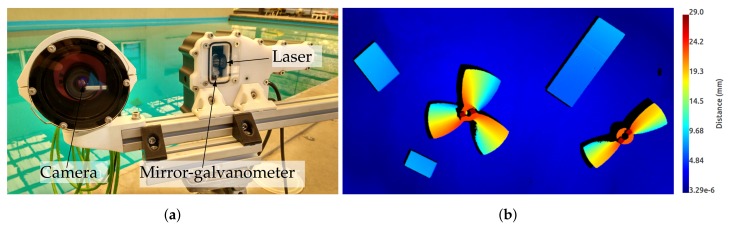
The laser scanner mounted on a tripod (**a**) and an example of the point cloud gathered with it coloured according to each point’s distance to the background (**b**).

**Figure 2:**
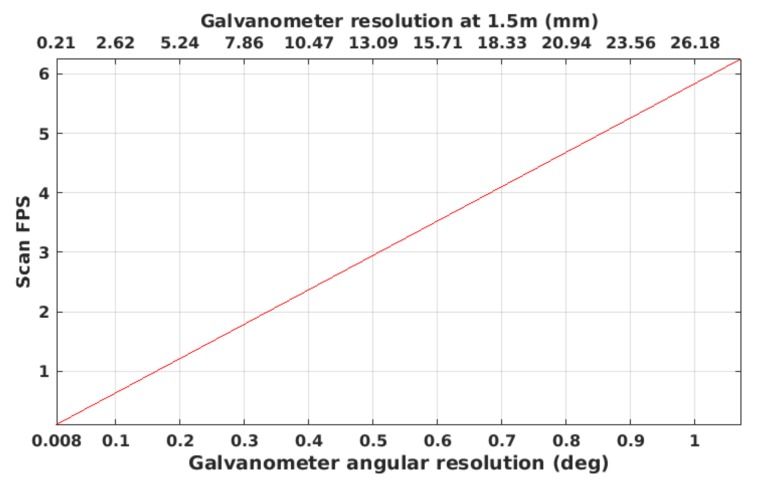
Laser scanner frame rate.

The proposed laser scanner consists of a galvanometer steering a mirror, a laser line projector, a camera and housing with two sealing viewports, one for the camera and one for the laser. The laser is projected into the mirror and reflected to the scene through the laser viewport. The galvanometer actuates the mirror in a way that for each image, the laser line is projected onto a different part of the scene. The camera and the galvanometer are synchronized electronically in a way that, for each galvanometer position, the camera is triggered once. This produces an image on the camera that contains only one single line. By accumulating all the laser points computed for each mirror position during a full scan, the sensor can produce a 3D point cloud similar to the one obtained by stereo imaging or depth cameras (see Figure 1). The proposed laser scanner can operate from 0.5–5 m, depending on water visibility conditions and surface light absorption, at a frame rate from 0.09 Hz at full resolution (0.008 degrees of galvanometer rotation between projected lines) up to 6 Hz at lower resolution (see Figure 2). The combination of the hardware elements (camera and galvanometer-mirror) in conjunction with the elliptical cone light representation (see Section 4.1.5) makes this scanner faster than state of the art systems.

**Figure 3:**
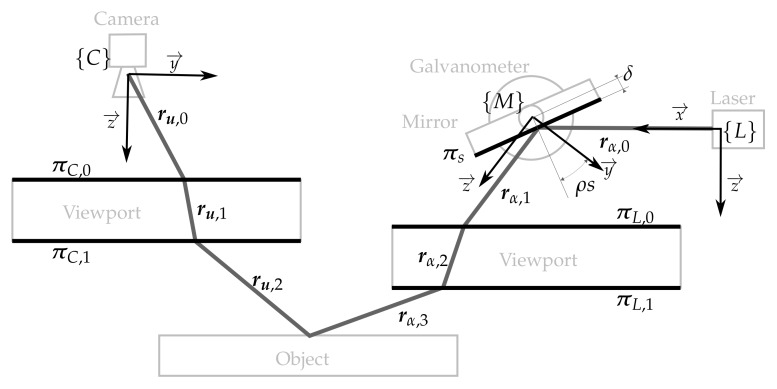
Illustration with the different elements of the sensor (light grey), the elements of the mathematical model (black) and the light path (dark grey).

The laser scanner model that describes the sensor is depicted in Figure 3 and consists of: (1) the laser pose with respect to the camera ${}^{\left\{C\right\}}t_{\left\{L\right\}}={\left[t_{x}\;t_{y}\;t_{z}\;t_{\phi }\;t_{\theta }\;t_{\psi }\right]}^{T}$; (2) the mirror-galvanometer model $\Pi =\{\rho ,\delta ,{}^{\left\{C\right\}}t_{\left\{M\right\}}\}$ where ρ is the galvanometer mechanical step (the rotation of the mirror plane around the $\vec{x}$ axis of the mirror frame {M} can be computed using the step *s* and ρ), δ is the mirror surface distance to the mirror rotation axis and ${}^{\left\{C\right\}}t_{\left\{M\right\}}$ is the transformation from the camera frame to the mirror frame; (3) the laser viewport $\Omega_{L}=\left\{\pi_{L}\;t_{L}\right\}$ with central plane $\pi_{L}$ and thickness $t_{L}$ representing the two laser viewport interfaces $\pi_{L,0}$ and $\pi_{L,1}$; (4) the camera model with the pin-hole parameters $\left\{f_{x},f_{y},c_{x},c_{y}\right\}$ and the radial $\left\{k_{1},k_{2},k_{3}\right\}$ and tangent $\left\{p_{1},p_{2}\right\}$ distortion parameters; and (5) the camera viewport $\Omega_{C}=\left\{\pi_{C}\;t_{C}\right\}$ representing the two camera viewport interfaces $\pi_{C,0}$ and $\pi_{C,1}$.

Given this model, the path that a ray of light emitted at an angle α around the $\vec{z}$ direction of {L} (i.e., $r_{\alpha ,0}$) can be computed using reflection and refraction. The ray $r_{\alpha ,0}$ is reflected onto the mirror surface $\pi_{s}$ at the angular step *s* producing $r_{\alpha ,1}$. This is then refracted on both sides of the laser viewport. First on $\pi_{L,0}$ producing $r_{\alpha ,2}$ and then on $\pi_{L,1}$ producing $r_{\alpha ,3}$, which is projected onto the scene. The light path on the camera side is traced the opposite way from its actual direction. Therefore, the light path is computed from the camera to the scene. The ray $r_{u,2}$, the reflection of $r_{\alpha ,3}$ on the scene that lit the pixel u, is traced from the camera ray $r_{u,0}$. This ray is refracted on both sides of the camera viewport, first with $\pi_{C,0}$ producing $r_{u,1}$ and, then, with $\pi_{C,1}$ producing $r_{u,2}$.

Given these two light paths, the 3D point associated with the laser detection on the camera image at the pixel u can be computed by intersecting the two rays $r_{u,2}$ and $r_{\alpha ,3}$. For a camera ray $r_{u,2}$, there must be only one laser ray $r_{\alpha ,3}$ that intersects with the camera ray, otherwise, the pixel u could not be lit by the laser. To find which α produces the intersection, a minimization process over the distance between the two rays is done. Computing the 3D points using this technique is time consuming because of the minimization process. In [17], the authors present results proving that an elliptical cone surface represents the light after the flat viewport refraction (i.e., the elliptical cone contains the rays $r_{\alpha ,3}\forall \alpha \in \left[\alpha_{0},\alpha_{1}\right]$ where $\alpha_{1}-\alpha_{0}$ is the aperture of the laser line). Then, the 3D points can be computed by intersecting the ray $r_{u,2}$ with the elliptical cone associated with the mirror position *s*. Using the elliptical cone offers a clear advantage when compared with the ray intersection technique because the ray-cone intersection has a closed form solution, and there is no iteration process as opposed to the parameter α triangulation. However, this requires an extra calibration step because the elliptical cone for each angle *s* at which the scanner will work has to be computed (see Section 4.1.5).

### 3.2. Fixed-Based Underwater Manipulator System

**Figure 4:**
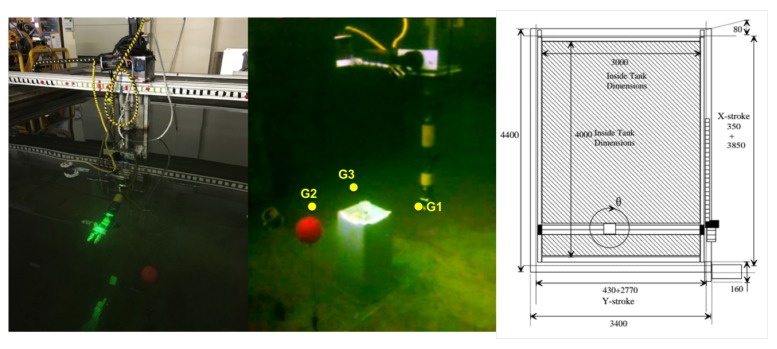
Water tank with the six DoF robot arm mounted on the head of the Cartesian manipulator (**left**); underwater scene with the three way-points defined for the experiment (centre; see Section 5.2); sketch of the Cartesian manipulator (**right**; units in mm).

The first system used to demonstrate the underwater laser capabilities is a redundant eight DoF robot manipulator (see Figure 4). This manipulator consists of a two DoF Cartesian manipulator and a six DoF robot arm attached to its head. The laser scanner was mounted on a pan and tilt attached to the head of the Cartesian manipulator. A brief description of the components follows.

#### 3.2.1. Cartesian Manipulator

The Cartesian manipulator has three DoF (*X*, *Y* and *Z*) with a length of 3.85 m, 2.77 m and 0.3 m, respectively. Given the small length of the *Z* axis, only the other two were used. At the end of the *Z* axis, there is a mechanical head where we attached the robot arm and the laser scanner previously described. The robot controller accepts simple commands to drive the head towards a desired position or to follow desired trapezoidal velocity profiles, amongst others. Moreover, a Robot Operating System (ROS) wrapper developed at the Ocean Systems Laboratory (OSL) was already available, simplifying the integration process with the other systems.

#### 3.2.2. 6 DoF Robotic Arm

The robot arm used was an HDT-Global Adroit-M arm with six DoF equipped with a dexterous hand with three fingers and four DoF. This electrically-driven robot arm rated for a 100-m depth has a length of around 1 m, weighs 10 kg in air and may lift up to 16 kg in air. The arm control software works in ROS providing interfaces to control it in joint or end-effector space. The arm can be controlled in position, velocity and torque.

### 3.3. Underwater Vehicle Manipulator System

**Figure 5:**
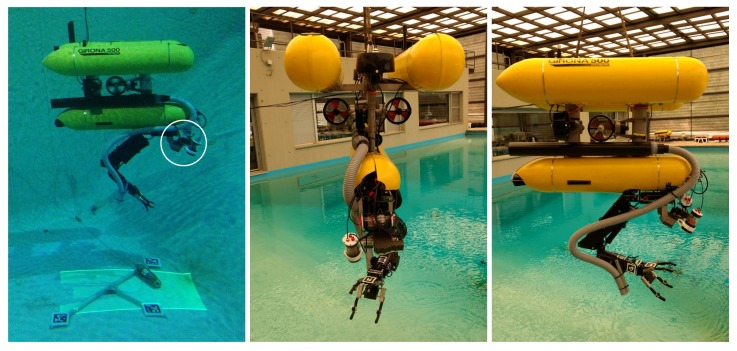
Girona 500 AUV with the four DoF robotic arm and the laser scanner. On the left, the robot is scanning an amphora, and the scanner is highlighted with a white circle. On the centre and on the right, the robot is ready for deployment.

The second system used to demonstrate the underwater laser capabilities for manipulation purposes is a free-floating redundant eight DoF robot manipulator (see Figure 5). In this case, the system consists of the Girona 500 AUV [18] and an ECA/CSIP 5E lightweight underwater arm. The laser scanner was mounted fixed on the Girona 500 payload area.

#### 3.3.1. Girona 500 AUV

The Girona 500 AUV is a compact and lightweight vehicle rated for a 500-m depth with survey and intervention capabilities. The overall dimensions of the AUV are 1 m in heigh, 1 m in width and 1.5 m in length with a weight under 200 kg. The two upper hulls contain the flotation, while the lower hull contains more heavy elements such as batteries or the payload area. This makes Girona 500 AUV an especially good vehicle for intervention purposes because its difference between the flotation centre and mass centre provides the AUV with a very good passive stability. With the current thruster configuration, the AUV has four DoF. The vehicle can be controlled in position, velocity and force and is integrated with ROS.

#### 3.3.2. Four DoF Robotic Arm

The robot arm used was an ECA/CSIP Light-Weight ARM 5E. This underwater electrical arm is composed of four revolute joints with reachable distances of 1 m and is equipped with a one DoF gripper. The arm is composed of aluminium partially covered with foam and rated to a 300-m depth. It weighs 29 kg in air, which decreases to 12 kg underwater, and can lift up to 12 kg at full reach. This robotic arm is integrated with ROS, has interfaces to its joint positions and can be controlled in joint or end-effector space.

## 4. Calibration

This section gives an overall view of the calibration process of the laser scanner presented in Section 4.1. Then, Section 4.2 proposes a calibration method to integrate the laser scanner and the robotic arm so they can be used together in the advanced manipulation tasks presented in Section 5.

### 4.1. Laser Scanner Calibration

The laser scanner calibration process consists of four steps used to estimate the elements of the model: (1) in-air camera calibration (see Section 4.1.1); (2) in-air laser calibration (see Section 4.1.2); (3) camera viewport calibration (see Section 4.1.3); and (4) laser viewport calibration (see Section 4.1.4). Moreover, instead of using a plane, an elliptic cone is used to model the laser light corresponding to each mirror position (see Section 4.1.5), to deal with the beam distortion induced by the flat viewport. The calibration method presented here is further detailed in [19].

#### 4.1.1. In-Air Camera Calibration

The camera calibration process uses multiple views of a chessboard pattern to produce pairs of 2D image points (pixels detections of each chessboard corner) with the 3D object points (the actual 3D position of each chessboard corner with respect to the chessboard frame in metric units). These pairs of points for different views are used to fit the pin-hole camera model, as well as the lens distortion model. In this work, we use a pin-hole camera model $\left\{f_{x},f_{y},c_{x},c_{y}\right\}$ with a radial $\left\{k_{1},k_{2},k_{3}\right\}$ and tangent $\left\{p_{1},p_{2}\right\}$ distortion model such as the one presented in [20]. During the estimation of these parameters, the camera position with respect to the object is also estimated for each image of the object. The camera calibration is done using the Open Source Computer Vision (OpenCV) library [21] built-in functions.

#### 4.1.2. In-Air Laser Calibration

**Figure 6:**
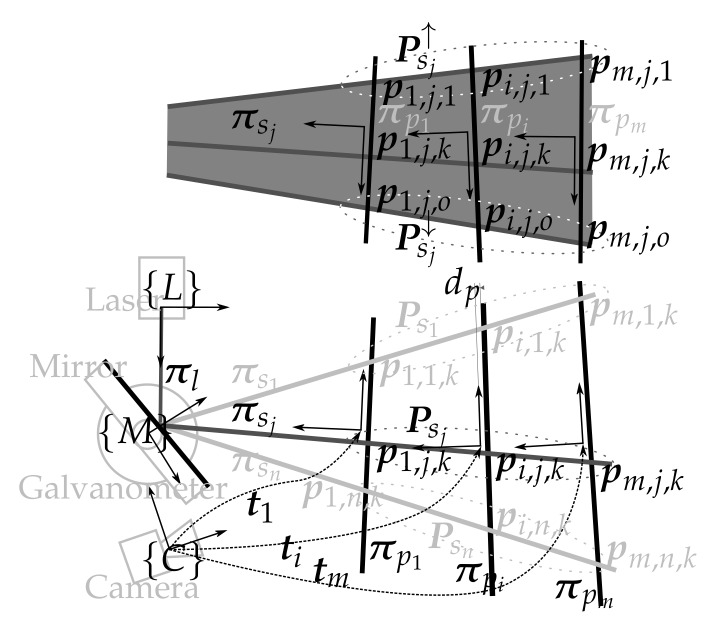
Illustration with all the elements from the air laser calibration. The top part of the figure shows the vertical view of the laser plane $P_{s_{j}}$. The bottom part shows the top view of the laser scanner and the calibration planes.

Using the in-air camera calibration and pairs of laser points retro-projected in the image plane (2D points) and the corresponding 3D laser points (laser projections onto a calibration plane), it is possible to estimate the mirror-galvanometer model $\Pi =\{\rho ,\delta ,{}^{\left\{C\right\}}t_{\left\{M\right\}}\}$ and the laser pose ${}^{\left\{C\right\}}t_{\left\{L\right\}}$. The calibration data are generated by projecting the laser light at *n* different galvanometer-mirror steps ($s=\{s_{1},\cdots ,s_{j},\cdots ,s_{n}\}$) onto a planar surface $\pi_{p_{i}}$ for *m* camera relative poses $t_{i}$ ($T\;=\;\{t_{1},t_{2},\cdots ,t_{m}|t_{i}=\left[t_{i,x},t_{i,y},t_{i,z},t_{i,\phi },t_{i,\theta },t_{i,\psi }\right]\}$) as shown in Figure 6. For each projection plane $\pi_{p_{i}}$, an estimate (${\hat{\pi }}_{p_{i}}$) of the plane, as well as an estimate of its pose (${\hat{t}}_{i}$) are computed using a visual pattern placed on it. Next, for each combination of estimated projection plane ${\hat{\pi }}_{p_{i}}$ and angle step $s_{j}$, the set of 3D plane points lit by the laser ($P_{i,j}=\left\{p_{i,j,1},p_{i,j,2},\cdots ,p_{i,j,o}|p_{i,j,k}=\left[p_{x}\;p_{y}\;p_{z}\right]\right\}$) is computed by intersecting the ray that passes through each laser detection pixel on the image plane (2D point), with the estimate ${\hat{\pi }}_{p_{i}}$ of the corresponding projection plane. Each one of the calibration pairs of points belongs at the same time to two groups: (1) the group of points reconstructing the projection plane $P_{\pi_{p_{i}}}={⋃}_{j=1}^{n}P_{i,j}$ (all the points gathered within one scan by steering the mirror along its complete range, scanning a projection plane fixed at a certain pose); and (2) the group of points of a specific mirror position $P_{s_{j}}={⋃}_{i=1}^{m}P_{i,j}$ (all the points gathered with the mirror positioned at a certain angle, for all the different poses of the projection plane).

The most basic in-air calibration consists of fitting a plane ${\hat{\pi }}_{s_{j}}$ amongst the 3D points of each mirror position $s_{j}$. With these planes, the first estimation of the mirror-galvanometer model can be computed. The rotation axis of the galvanometer can be computed by averaging all the direction vectors of the intersection line between two consecutive planes ${\hat{\pi }}_{s_{j}}$ and ${\hat{\pi }}_{s_{j+1}}$. This axis defines the $\vec{x}$ direction of the mirror-galvanometer reference frame. The $\vec{z}$ direction is the vector perpendicular to the $\vec{x}$, as well as to the normal of the first plane ${\hat{\pi }}_{s_{0}}$. The $\vec{y}$ is perpendicular to $\vec{x}$ and $\vec{z}$. With these three directions defined, the rotation part of ${}^{\left\{C\right\}}t_{\left\{M\right\}}$ is estimated. The translational part can be computed by averaging the points of the intersecting lines of the planes ${\hat{\pi }}_{s_{j}}$ and ${\hat{\pi }}_{s_{j+1}}$ and the plane z=0. The galvanometer mechanical step ρ can be computed by averaging the angle between all the pairs of planes ${\hat{\pi }}_{s_{j}}$ and ${\hat{\pi }}_{s_{j+1}}$. With the first estimation of the Π model, the original laser plane $\pi_{l}$ that generated each one of the $\pi_{s_{j}}$ planes for each mirror position $s_{j}$ can be estimated (${\hat{\pi }}_{l}$) by reflecting each laser plane ${\hat{\pi }}_{s_{j}}$ on the mirror and averaging all the reflected planes. With this simplified model for each 3D point, an error can be defined by intersecting the camera ray associated with the 3D point and the reflection of the laser plane ${\hat{\pi }}_{l}$ on the mirror at the corresponding position. This error can then be minimized to better estimate this sensor model.

The final part of the in-air laser calibration is estimating the laser focal point, in other words the laser pose ${}^{\left\{C\right\}}t_{\left\{L\right\}}$, which will be on ${\hat{\pi }}_{l}$. For this purpose, each end point of the projected lines is grouped taking into account its mirror position and to which line extreme it corresponds. Therefore, for each galvanometer-mirror position, two groups are created $P_{s_{j}}^{↑}$ and $P_{s_{j}}^{↓}$ that contain as many points as relative positions of the laser scanner with respect to the projection plane. Each group of points represents the reflection of the two extreme laser rays on the mirror at step $s_{j}$, where $P_{s_{j}}^{↑}$ is the top and $P_{s_{j}}^{↓}$ the bottom one. The ray can be computed by fitting a line for each group of points. Then, each fitted ray is reflected with its corresponding mirror position. The laser focal point is computed by finding the point on the plane ${\hat{\pi }}_{l}$ that is closer to all the reflected rays. Finally, the laser orientation is fixed by setting the $\vec{z}$ direction coincident with the normal of the plane ${\hat{\pi }}_{l}$, the $\vec{y}$ direction perpendicular to the $\vec{z}$ and the normal of the plane $\pi_{s_{0}}$ and the $\vec{x}$ direction perpendicular to $\vec{y}$ and $\vec{z}$.

#### 4.1.3. Camera Viewport

**Figure 7:**
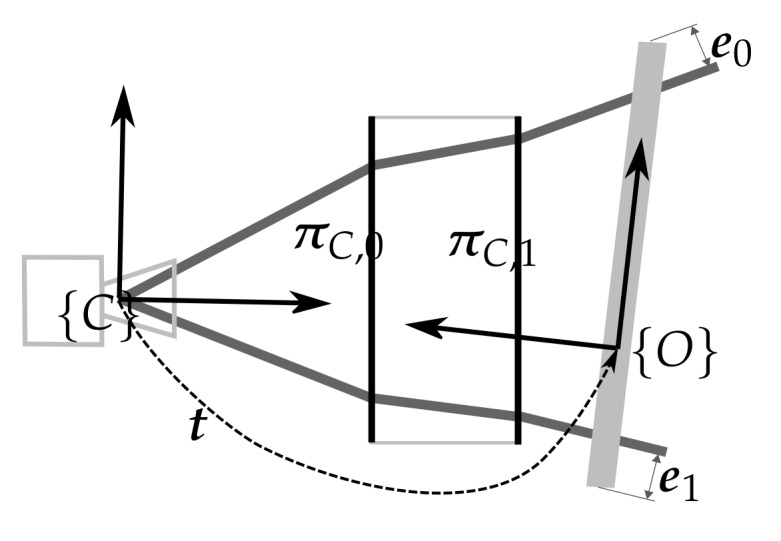
Illustration of the camera viewport calibration error for an object with two 3D points.

In this calibration step, the algorithm uses multiple underwater views of a chessboard pattern to produce pairs of 2D image points and the 3D object points, similar to the data used in Section 4.1.1. Given the camera viewport $\Omega_{C}$ and the transformation of the object to the camera ${}^{\left\{C\right\}}t_{\left\{O\right\}}$, an error can be defined between the ray that passes through the detected pixel and is refracted on both sides of the viewport and the object point. Using multiple views of the object, a minimization problem can be defined and optimized to estimate the camera viewport $\Omega_{C}$ and all the object to camera transformations. Figure 7 presents an illustration of the error computation for one position. In this illustration, the object has two 3D points (the two end points of the line). The ray that passes through each one of the pixels corresponding with the end points of the object is refracted on both viewport interfaces $\pi_{C,0}$ and $\pi_{C,1}$. Then, the error $e_{0}$ and $e_{1}$ between each object end point and its associated ray is computed.

#### 4.1.4. Laser Viewport

**Figure 8:**
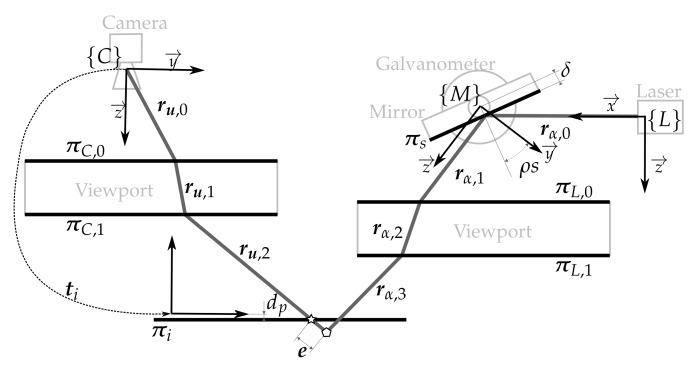
Illustration of the laser viewport calibration error. The star represents the point computed by intersecting the camera ray with the object while the pentagon represents the point computed intersecting the camera ray with the laser ray. Note that the laser ray $r_{\alpha ,3}$ and the camera ray $r_{u,2}$ do not intersect in the projection plane $\pi_{i}$ because the laser viewport is not estimated properly.

The laser viewport calibration uses the same type of data as the in-air laser calibration (see Section 4.1.2), but in this case, the plane and the sensor are underwater. For each view of the projection plane, the same minimization process used in the camera viewport calibration (see Section 4.1.3) can be used to estimate the relative pose $t_{i}$ of the camera and the pattern by setting the camera viewport $\Omega_{C}$ as constant. Then, each 3D point associated with each 2D image point of the laser can be computed by refracting the ray that passes through the image point on both sides of the camera viewport $\Omega_{C}$ and intersecting it with the projection plane (see the star in Figure 8).

Given the laser viewport $\Omega_{L}$ and the rest of the sensor parameters already calibrated, a point can be triangulated by intersecting the camera ray $r_{u,2}$ and the laser ray $r_{\alpha ,3}$ (see the pentagon in Figure 8). Although the camera ray is known and computed from the camera intrinsic parameters and the camera viewport (elements from the sensor that have already been calibrated), there is no knowledge of the angle α of the laser ray that intersects the camera ray $r_{u,2}$. However, there is only one ray from the laser that lights up the camera pixel u associated with $r_{u,2}$. The angle α of this laser ray can be computed by minimizing the distance between the two rays $r_{u,2}$ and $r_{\alpha ,3}$. An error between this point and its associated original 3D points (see e in Figure 8) can be defined. Minimizing the total error of all the points leads to the laser viewport $\Omega_{L}$ estimation.

#### 4.1.5. Elliptical Cone Fitting

In [17], the authors presented results proving that an elliptical cone better represents the surface containing the laser light in the underwater medium when a flat viewport is used to seal a laser projector such as the one of the laser scanner. The advantage of such a surface is that it represents well the refraction of the laser plane traversing a flat viewport, and a closed form solution for the ray-surface intersection exists, allowing for a quick triangulation computation.

Using the sensor model fitted during the calibration process, an elliptical cone is computed for each mirror-galvanometer position at which the sensor will operate. For each mirror position, rays are traced from the laser source, reflected on its corresponding mirror and refracted on both sides of the laser viewport. Then, points are sampled along the rays. These points represent the laser light in the underwater medium and are used to fit the elliptical cone for that mirror-galvanometer position.

$c\left(h,\beta \right)$ (see Equation (1)) being a cone with its vertex in the origin, its revolution axis along the $\vec{z}$ direction and aperture *a* and *b* in the $\vec{x}$ and $\vec{y}$ directions, respectively, the generic cone $g\left(h,\beta \right)$ can be computed using a transformation ${}^{\left\{W\right\}}t_{\left\{Q\right\}}$ (see Equation (2)). Defining $d\left(p,g\left(h,\beta \right)\right)$ as the distance between a point p and the generic elliptical cone $g\left(h,\beta \right)$, the parameters *a*, *b* and ${}^{\left\{W\right\}}t_{\left\{Q\right\}}$ minimizing the total addition of the distances of the points sampled on the rays created with the sensor model can be computed. Therefore, the generic cone that represents the laser projected light for that specific mirror-galvanometer position is computed and can be later used for on-line triangulation.

(1) $$c\left(h,\beta \right)={\left[\begin{array}{ccc} a\;h\;\cos\left(\beta \right) & b\;h\;\sin\left(\beta \right) & h \end{array}\right]}^{T}$$

(2) $$g\left(h,\beta \right){=}^{\left\{W\right\}}t_{\left\{Q\right\}}⊕c\left(h,\beta \right)$$

### 4.2. Laser Scanner-Arm Calibration

The calibration process consists of finding the relative poses of the different elements of the system so it becomes possible to relate their respective frames. In the case of the AUV equipped with a four DoF manipulator, the scanner was mounted fixed with respect to the manipulator arm (see Section 3.3). In the case of the eight DoF manipulator, the scanner was mounted on a pan and tilt unit whose base was fixed with respect to the base of the robotic arm (see Section 3.2).

To be able to estimate the relative pose of the arm end-effector with respect to the laser scanner, a marker was placed on the end-effector. This marker can be easily located (${}^{\left\{S\right\}}t_{\left\{M\right\}}$) with respect to the scanner’s camera using simple state of the art computer vision techniques [22].

**Figure 9:**
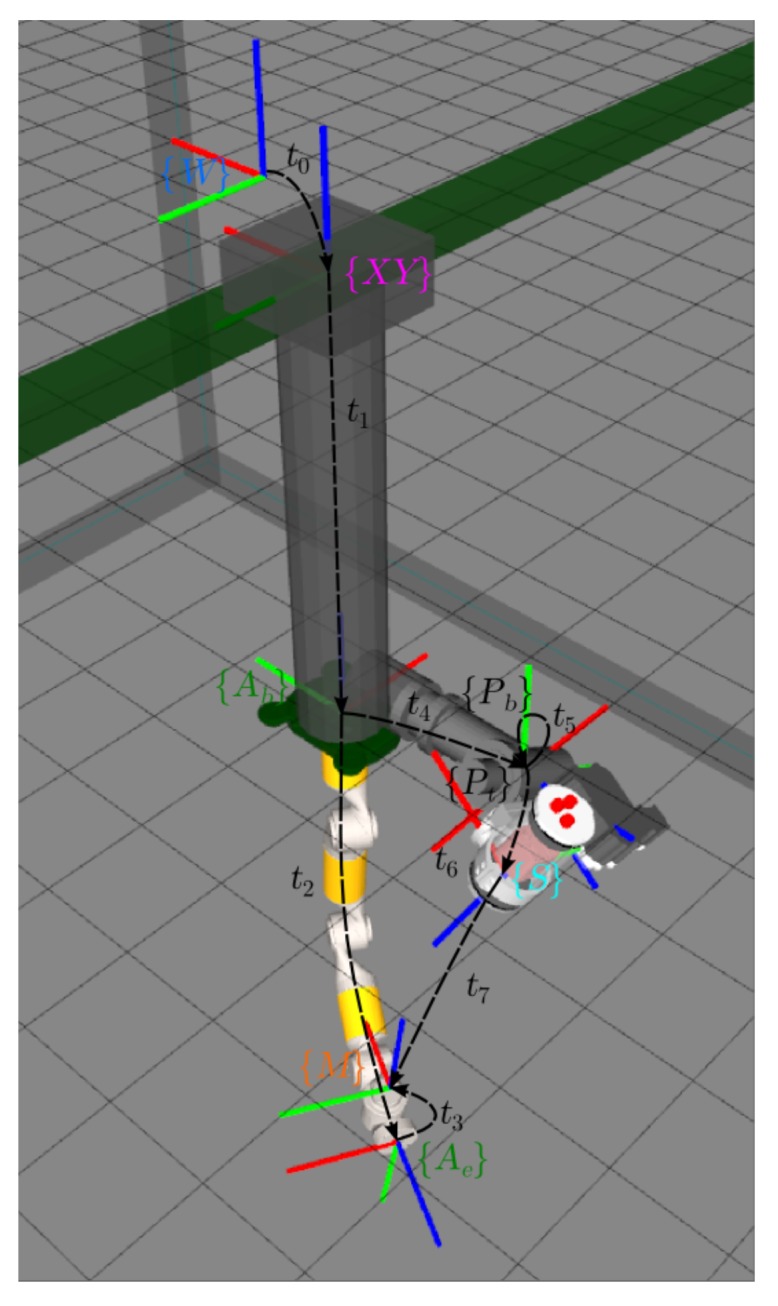
Schematics of the frames involved in the calibration process of the eight DoF manipulator. Each block/actuator has a different colour: {*W*} (blue) is the world reference frame; {XY} (pink) is the position of the plotter head; $\left\{A_{b}\right\}$ is the base; $\left\{A_{ee}\right\}$ (green) is the end-effector of the robotic arm; {M} (red) is the frame of a marker used for the calibration; $\left\{P_{b}\right\}$ is the base; $\left\{P_{t}\right\}$ (black) is the rotated axis of the pan and tilt actuator; and finally, {S} (blue) is the frame of the laser scanner. Each one of the $t_{i}$ represents the transformation between the corresponding reference frames.

In Figure 9, two kinematics chains linking $\left\{A_{b}\right\}$ and the marker {M} are found: (1) $\left\{A_{b}\right\}\;\to \;\left\{A_{ee}\right\}\;\to \;\left\{M\right\}$ defined by the transformations $t_{2}={}^{\left\{A_{b}\right\}}t_{\left\{A_{ee}\right\}}$ and $t_{3}={}^{\left\{A_{ee}\right\}}t_{\left\{M\right\}}$; and (2) $\left\{A_{b}\right\}\to \left\{P_{b}\right\}\to \left\{P_{t}\right\}\to \left\{S\right\}\to \left\{M\right\}$ defined by the transformations $t_{4}={}^{\left\{A_{b}\right\}}t_{\left\{P_{b}\right\}}$, $t_{5}={}^{\left\{P_{b}\right\}}t_{\left\{P_{t}\right\}}$, $t_{6}={}^{\left\{P_{t}\right\}}t_{\left\{S\right\}}$ and $t_{7}={}^{\left\{S\right\}}t_{\left\{M\right\}}$. For a given configuration of the arm and the pan and tilt unit, $t_{2}$ and $t_{5}$ are known due to the feedback of the arm and the pan and tilt. Moreover, $t_{7}$ can be estimated using computer vision techniques, as was done in the calibration section (see Section 4.1.4). Hence, the only unknown transformations are $t_{3}$, $t_{4}$ and $t_{6}$, and the three of them are constant.

Moving the end-effector, as well as the pan and tilt along several poses for which the visual mark is visible by the scanner camera, every time that the marker is detected, four points referenced to the marker frame {M} can be added to the set of point used to compute the transformation $t_{7}$. Since there are two kinematics chains to link the position of {M} with $\left\{A_{b}\right\}$, we can define an error on each of these four points by subtracting their positions in the arm base frame $\left\{A_{b}\right\}$ computed through the two different kinematics chains:

(3) $$e_{i}=t_{2}⊕t_{3}⊕p_{i}-t_{4}⊕t_{5}⊕t_{6}⊕t_{7}⊕p_{i}$$

**Figure 10:**
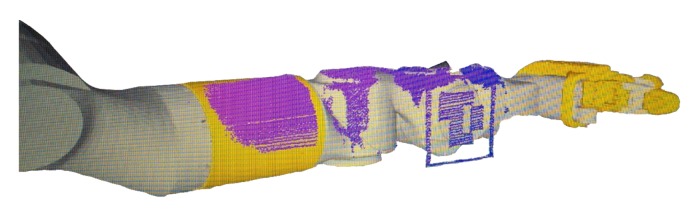
Eight DoF manipulator calibrated. It can be seen how the sensed point cloud overlays the six DoF arm.

With this point error, we can define the following non-linear least squares problem:

(4) $$\left[t_{3},\;t_{4},\;t_{6}\right]=\underset{\left[t_{3},\;t_{4},\;t_{6}\right]}{\mathrm{argmin}}\sum_{k}\sum_{i=0}^{3}e_{i,k}^{T}e_{i,k}$$

where *k* is the set of different configurations of arm pan and tilt where each marker was observed. In the same way, the error can be defined in the AUV with a four DoF manipulator to find the two transformations unknown in that system: ${}^{\left\{A_{b}\right\}}t_{\left\{S\right\}}$ and ${}^{\left\{A_{ee}\right\}}t_{\left\{M\right\}}$. Figure 10 shows a very simple visual confirmation of a correct calibration result. The scanner was used to image the fore arm and the wrist of the eight DoF manipulator. The figure shows how the estimated 3D point cloud overlays the manipulator 3D model, providing an idea about the calibration accuracy.

## 5. Experiments and Results

In this section, first an experiment to assess the sensor accuracy is presented followed by the two experiments involving each one of the mechatronics systems. In the two experiments where the sensor is integrated with a manipulator system, it is shown how the scanner allows one to implement an advanced underwater manipulation task beyond the state of the art of current underwater intervention systems, illustrating the utility of the proposed system.

### 5.1. Sensor Accuracy

**Figure 11:**
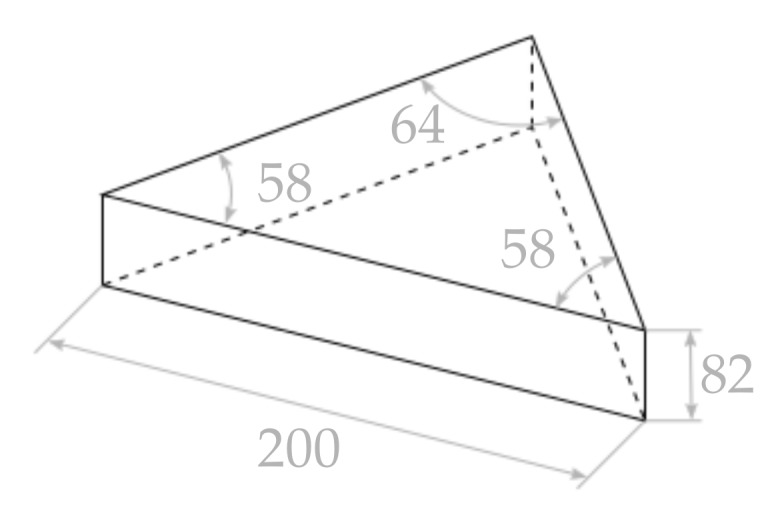
Sketch of the triangular prism used for the experiment. The units are millimetres for the distances and degrees for the angles.

**Figure 12:**
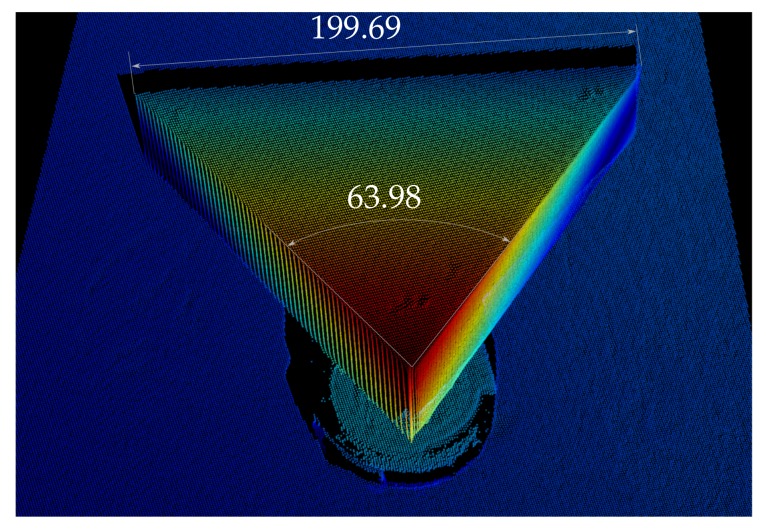
Partial view of a reconstructed area. The cloud has been coloured according to each point’s distance to the background for easier interpretation. The units are millimetres for the distance and degrees for the angle.

This experiments consists of scanning a triangular prism to assess how accurately the 3D points represent the real object. The prism consist of two 58 degree angles separated 200 mm and a third angle of 64 degrees (see Figure 11). In the experiment, it is positioned in a way that both faces with a respective angle of 64 degrees, as well as the 200-mm edge are clearly visible. Then, this angle and longitude are measured for each one of the reconstructions by manually selecting points in the cloud. In the case of the edge of the prism, two points are selected, while in the case of the angle, an area is selected on each face, and the angle between the two normal vectors of the two selected areas is computed. Figure 12 presents one of the evaluated scans gathered underwater. In the figure, it can be seen how the error of the measurement of the edge is below 1 mm and the error of the measured angle is below 0.1 degrees. This has been done for a total of 10 different positions of the prism. The average error for the 10 different positions of the is 0.53 mm with a standard deviation of 0.27 mm. The angle mean error is 0.072 degrees with a standard deviation of 0.015 degrees.

### 5.2. Motion Planning of a Fixed-Base Manipulator in the Presence of Unknown Fixed Obstacles

In this experiment MoveIt! [23] is used to plan and control the trajectory of the eight DoF underwater manipulator. MoveIt! is a mobile manipulation software making available state of the art techniques implemented in standard libraries like the Kinematics and Dynamics Library (KDL), the Open Motion Planning Library (OMPL) [24] the Fast Collision Check Library (FCL) [25] and the OCTOMAP [26]. The motion planning problem tackled falls under the category of an unknown/time-invariant environment, given that the robot surroundings are initially unknown to the system, but populated only with static obstacles that will be gradually added to the system map when scanned during the arm motion.

For this work, the RRT-connect algorithm [27] was chosen amongst the wide variety of planning algorithms already available at the OMPL. This planner uses the free and occupied space from an OCTOMAP, which is constantly updated using the laser scanner 3D data. Therefore, it is able to plan an obstacle-free path. Moreover, the plan is constantly being checked with the latest OCTOMAP data. In the eventual case that the already planned path collides with a region that becomes occupied when a new obstacle is discovered during the motion, it is aborted. In this case, a new path is computed to reach the final goal without colliding with the new discovered object. This implementation is similar to our previous work in [28], but in this case, instead of dealing with an a priori known map, the laser scanner is used to map the environment in real time during the arm motion.

The experiment consisted of moving the end-effector of the eight DoF manipulator cyclically through three way-points located in a water tank within an area cluttered with unknown obstacles. At the beginning of the experiment, the occupancy map was empty, allowing the trajectories to be planned everywhere in the robot work space due to the absence of obstacles. During the execution of the experiment, the map was constantly updated with the new 3D points of the laser scanner. If the computed trajectory collided with some newly discovered object, the execution was stopped, and a new trajectory towards the goal was computed. The experiment was performed in the water tank of the OSL at Heriot Watt University. A big plastic container (box), as well as two small obstacles (a cup and a sphere) were placed close to each other separating the direct line between the three way-points. Figure 4 shows the environment along with the goals.

**Figure 13:**
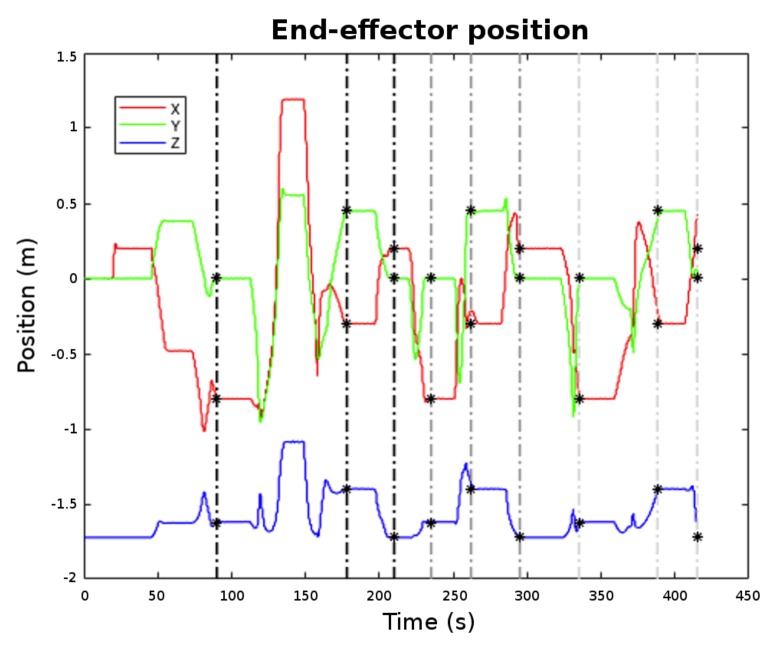
End-effector position with different way-points marked.

**Figure 14:**
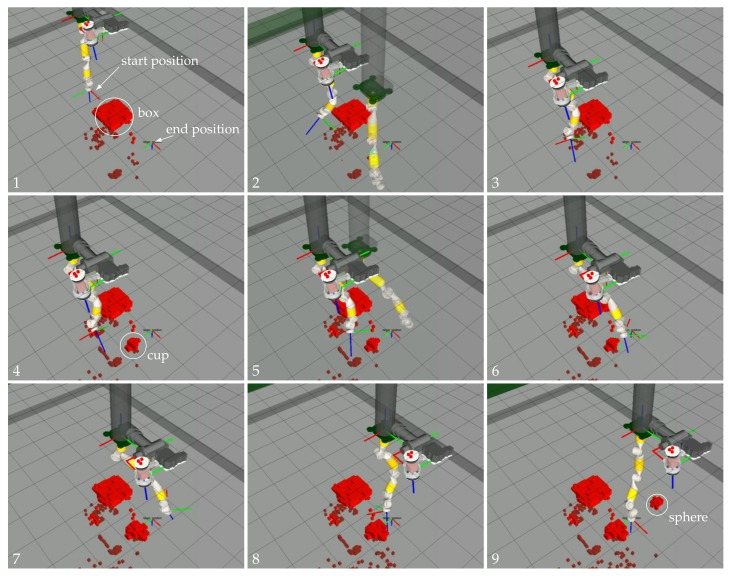
Sequence (**1**–**9**) of one of the trajectories of the eight DoF manipulators from Goal 1 to Goal 2. The shadow robot represents the simulation of the robot following the computed trajectory.

Figure 13 shows the end-effector motion along time. The goal way-points are marked with black stars. It can be appreciated from the graph that the arm was reaching each requested goal with accuracy. The end-effector attitude is omitted in Figure 13 as it was kept constant for all three goals during the mission (${\left[\phi \;\theta \;\psi \right]}^{T}={\left[0\;2\pi \;0\right]}^{T}$). Note that the end-effector trajectory changed along the cycles due to the random nature of the RRT-connect path-planning algorithm from one side and due to the fact that during the transit, the environment was being discovered, provoking, sometimes, a trajectory re-planning. Figure 14 shows the arm motion through the working area while it transits for the first time from Way-point 1 to Way-point 2. There, it can be seen how the trajectory stops when the OCTOMAP updates the voxels corresponding to the cup (Frame 4) and how it re-plans to reach Way-point 2 without colliding with the environment. Please see the Supplementary Material for the video of the overall experiment.

### 5.3. Object Grasping with a UVMS

In this experiment, the Girona 500 AUV was equipped with the ECA/CSIP Light-Weight ARM 5E and controlled using the task priority framework. The task priority approach is out of the scope of this publication; please see [15] for a detailed description of a similar control system. The experiment consisted of picking up an amphora from the bottom of a water tank, in this case, free of obstacles. During the experiment, the amphora position was computed for every newly available data, providing continuous feedback to the task priority control on the grasping pose. Please see [29] for a description of the grasping pose computation from a point cloud, which is out of the scope of this publication. To avoid end-effector and object collisions, the grasping procedure consisted on two stages. First, the UVMS end-effector was moved to a pose aligned with the grasping pose, but at a safe distance along the $\vec{z}$ of the grasping pose (approximately 20 cm). Then, the task priority moved the UVMS end-effector to the grasping pose. Finally, when the end-effector reached the grasping pose, the claw was closed, and the UVMS was sent to surface. The experiment was done in the context of the MERBOTS project [30], were two underwater robots cooperate during a semi-autonomous intervention operation. The visual markers placed on the floor of the water tank were used for accurate visual-based navigation, as well as to allow both robots to accurately navigate within the same frame of reference, not being relevant for the the experiment described here.

**Figure 15:**
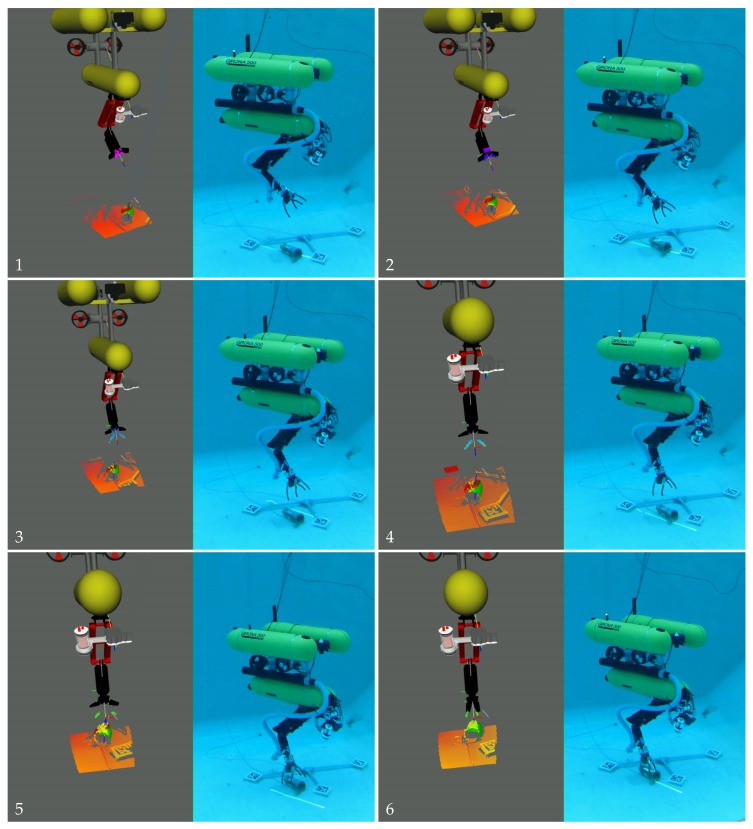
Sequence of images (**1**–**6**) of the 3D visualizer alongside the real scenario while the robot approaches and grasps the amphora.

It can be seen in Figure 15 how the green mesh corresponding to the detected position of the amphora, using a ground-truth marker located on it, corresponds to the amphora position in the point cloud. Moreover, in this same figure, the approach and grasping process is presented in a sequence to demonstrate that the 3D point cloud was being updated in real time during the complete grasping operation (note that the misalignment between the sensed jaw of the robotic arm and the point cloud is because the opening of the jaw is not properly modelled in the 3D viewer). Please see the Supplementary Materials for the video of the overall experiment.

## 6. Conclusions and Future Work

This paper has presented an underwater 3D laser scanner and its application to advanced underwater manipulation tasks using two different manipulation systems. The paper described the laser scanner and outlined its calibration method. Moreover, in order to integrate it with an underwater robot arm, a scanner-to-arm calibration method was also proposed and implemented. The sensor accuracy was tested using a known object reconstructed at different distances with measurement errors smaller than one millimetre and 0.1 degrees. Next, the system was demonstrated using and eight DoF fixed-base manipulation system following paths planned in real time. The arm was moved through an environment populated with fixed obstacles located at unknown positions that were discovered during the motion. A second demonstration was done using a UVMS to autonomously grasp an object from the bottom of a water tank. In both cases, the 3D information provided by the laser scanner was fundamental to complete the task and its accuracy proved to be suitable for the manipulation purposes.

Future work includes further work in correcting the internal error in the scan as a consequence of sensor motion. Increasing the sensor frame rate, this distortion will be reduced because the sensor movement during a scan will decrease. Another approach will be incorporating data from a navigation sensor (such as an Internal Navigation Systems (INS)) to be able to estimate the sensor motion during a scan. It also includes testing of the laser scanning with the state of the art methods for 3D object recognition, in order to introduce semantics for subsea intervention. Moreover, integrating this type of data and object recognition and pose estimation with underwater manipulation systems can help to push the underwater manipulation from the research level to an every-day used technique in the underwater industry.

## Supplementary Materials

The Supplementary Materials are available online at http://www.mdpi.com/1424-8220/18/4/1086/s1.
